# Wandering Spleen: A Medical Enigma, Its Natural History and Rationalization

**DOI:** 10.1007/s00268-012-1880-x

**Published:** 2012-12-13

**Authors:** Anita Magowska

**Affiliations:** History of Medical Sciences, Poznan University of Medical Sciences, ul. Przybyszewskiego 37A, 61-111 Poznan, Poland

## Abstract

**Introduction:**

Wandering spleen is a rare condition in which the spleen is not located in the left upper quadrant but is found lower in the abdomen or in the pelvic region because of the laxity of the peritoneal attachments. Many patients with wandering spleen are asymptomatic, hence the condition can be discovered only by abdominal examination or at a hospital emergency department if a patient is admitted to hospital because of severe abdominal pain, vomiting or obstipation.

**Methods:**

This article aims to provide a historical overview of wandering spleen diagnostics and surgical treatment supplemented with an analyses of articles on wandering spleen included in the PubMed database.

**Results:**

One of the first clinical descriptions of a wandering spleen was written by Józef Dietl in 1854. The next years of vital importance are 1877 when A. Martin conducted the first splenectomy and in 1895 when Ludwik Rydygier carried out the first splenopexy to immobilize a wandering spleen. Since that time various techniques of splenectomy and splenopexy have been developed.

**Conclusions:**

Introducing medical technologies was a watershed in the development and treatment of wandering spleen, which is confirmed by the PubMed database. Despite the increased number of publications medical literature shows that a wandering spleen still remains a misdiagnosed condition, especially among children.

## The development of knowledge referring to wandering spleen

Ayurveda, the classical Indian system of medical practice based on the humor doctrine, describes the spleen as “the root of the ducts which transport the blood” [1]. The ancient Greek humoral system of Hippocrates and Galen, in some ways analogous to that description, attributed to the spleen the role of an organ responsible for producing black bile, whose Greek name: μελανχολία, is the root of the English word *melancholia* [2]. Over hundreds of years, doctors, strongly influenced by the legacy of the humoral theory, successfully anchored the spleen in the pathogenesis of neurasthenia and hypochondria experienced by women, whereas women’s hysteria was explained as the result of wandering of the uterus. Even when advances in the field of morbid anatomy indicated the obvious absurdity of humoral theory, doctors were unable to free themselves from these stereotypical notions. It was as late as 1682 when an outstanding English physician, Thomas Sydenham (1624–1689), provided evidence for strangulation of the womb [3], whereas as recently as 1863, Józef Dietl (1804–1878) [4], an internationally acclaimed clinical doctor, wrote that a wandering spleen led women to experience hypochondria and that a wandering uterus caused hysteria. In 1908 John Duncan [5] expressed his belief that wandering organs, including a wandering spleen, are an expression of neurasthenia.

In 1653, Panoralus [6] for the first time described the spleen as a “ductless gland”. Next, in 1667 Van Horne recognized and described the phenomenon of a wandering spleen [7]. Even though post-mortem examinations were carried out more and more often in the seventeenth century, they did not provide any information as to how important the spleen could be for the human body. No wonder, then, that in 1725 Sir Richard Blackmore (1654–1729) [8], an English doctor and poet, questioned whether it is necessary to have a spleen for the human body to function normally.

In the nineteenth century the spleen still remained a medical enigma. The authors of German anatomy atlases, Robert Foriep (1804–1861), a doctor and an artist who followed Italian lithographers, and Theodor Richter (1824–1898), who was helped by a professional illustrator, did not pay much attention to the spleen. Interestingly, such a rare phenomenon as a wandering spleen kept arousing enormous interest among physicians. A wandering spleen resulted in the characteristic dullness of lung sounds on percussion and was proved by palpation. The greatest authorities in medical science widely described the diagnosis of a wandering spleen, including the Scottish doctor and philosopher John Abercrombie (1780–1844), who wrote “Researches on the Diseases of the Intestinal Canal, Liver and other Viscera of the Abdomen” (Edinburgh 1838) and the most prominent member of the Vienna School, Carl von Rokitansky (1804–1878), who described the phenomenon in the course-book “Lehrbuch der patologische Anatomie” (Textbook on Morbid Anatomy) (Vienna 1846) [9].

**Fig. 1:**
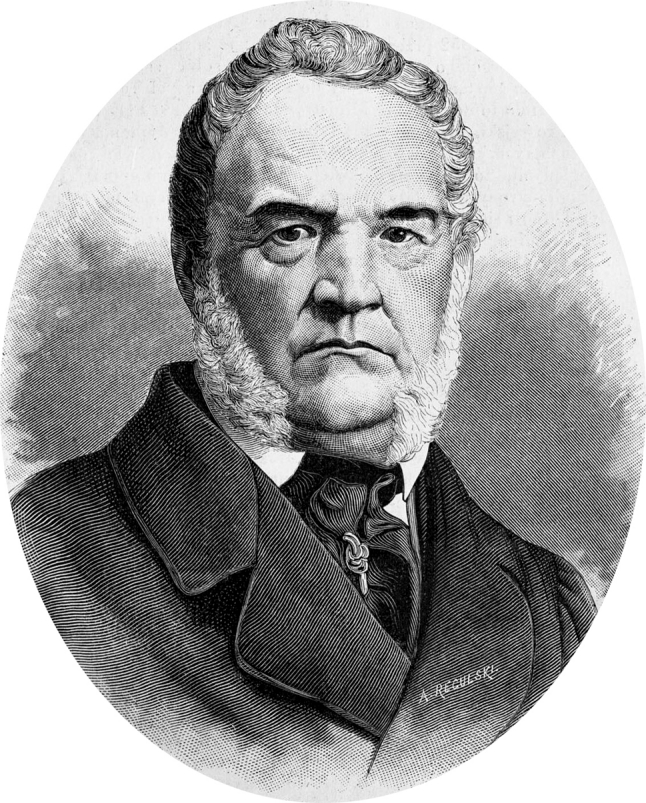
Józef Dietl (1804–1878), a Polish physician who described one of the first cases of a wandering spleen in a child (by courtesy of the Polish National Digital Archives)

One of the first case reports of a wandering spleen in a child was published in 1854 by the Polish physician Józef Dietl (Fig. 1) in the Polish journal “Pamiętnik Towarzystwa Lekarskiego Warszawskiego” (Diary of the Warsaw Medical Society) and in ”Wiener Medizinische Wochenschrift.” Two years later Dietl [10] included in the same periodical his next observations of a case related to a wandering spleen, yet this time he took the post mortem examination into consideration. In 1863 Dietl described a third case of a wandering spleen in “Przegląd Lekarski,” a journal he founded and edited himself. It was there that he indicated this condition to be life-threatening because it led to an extensive peritonitis and consequently death. He was one of the first doctors who stated that it was not a patients’ temperament but rather relaxation, extension, or the hypoplasia of splenic ligaments that made a spleen wander. He considered wandering spleen to be a condition present in women emaciated and exhausted by extensive work. He treated the condition by using quinine (he believed it decreased the size of the spleen and improved his patients’ mood) and an abdominal compression binder made of plain linen or rubber. Surgical removal of the spleen, recommended by Friedrich Kűchenmeister (1821–1890), was considered by Dietl [4] as definitely too risky. Still, he allowed the abdominal wall to be pierced with a knife in order to provoke limited inflammation and local adhesion.

In his historical study of surgery, Ricketts [8] cited Dietl’s article published in 1863 as a classical description of clinical wandering spleen complications. This text, however, did not gain as much publicity as a clinical description of a wandering kidney incarceration on the basis of which the eponym “Dietl’s crisis” was created [11]. By no means was it a coincidence that Dietl, who was both an internist and anatomopathologist, became interested in the symptoms of both a wandering kidney and a wandering spleen, even though in the interwar period doctors considered such symptoms a single clinical problem [12].

## Advances in spleen surgery

Before narcosis and antiseptic treatment had been introduced, the diagnosis of a wandering and/or enlarged spleen seldom if ever became an indication for organ removal. In 1549, Adrian Zacarelli for the first time had demonstrated removal of an enlarged spleen [13]. In the following centuries war wounds imposed on doctors the necessity for surgical treatment of the spleen. Its removal was considered necessary in cases of rupture or ulcerative stomach wounds [8], what in 1788 was first described by Giovanni Fantoni in “Opuscula Medica et Physiologica,” a work published in Geneva. Then, in 1855 Darmstadt, Julian Schultz completed the successful removal of a spleen protruding from a wound in the patient’s side [14]. The first fully documented and successful removal of a wandering spleen was carried out by Martin [15] in 1877 in Berlin. One year later Vincenz Czerny (1840–1916), in Heidelberg, made the next successful surgical removal of a wandering spleen [14].

**Fig. 2:**
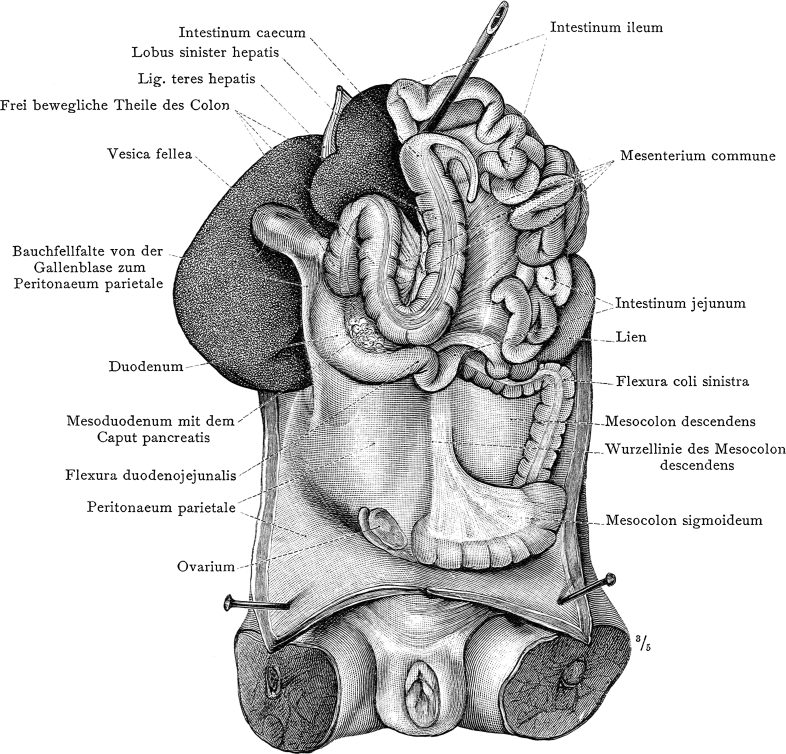
One of the first images of spleen, where it is marked as a separate organ with its own name ‘lien’, in “Anatomischer Atlas” by an Austrian anatomist Carl Toldt (1840–1920), (Berlin–Vienna 1906), the photo made by the author

To sum up, between 1855 and 1903 doctors conducted 360 splenectomies, 38.3 % of which resulted in the patients’ death from hemorrhage and shock (Fig. 2) [16]. It is worth stressing that in 1865–1875 up to 80 % of patients died after spleen removal [8]. Such a high mortality rate resulted from the fact that one of the first indications for splenectomy was not only an enlarged and/or wandering spleen but also evidence of leukemia [12]. To 1900, the mortality rate among patients with a removed spleen in the treatment of leukemia amounted to 87.7 % [16]. As late as the interwar period, all cases of splenomegaly, including conditions caused by malaria, kala-azar disease, leukemia, and anemia, or any unknown conditions, were treated by removing the spleen [12].

The verification of rules according to which patients were selected for splenectomy was made thanks to the analyses of cases described in the medical literature. These analyses were made inter alia by Thornton (1886) [14], Wells (1888) [17], Abell (1933) [18], Lahey, and Norcross (1948) [19]. Of 500 splenectomies carried out to 1930 at the Mayo Clinic, there were only two cases of wandering spleen [20]. The analysis of 93 cases of wandering spleen with torsion of the pedicle made by Abell in 1933 showed that 88 cases occurred in women, mostly of an age ranging from 21 years to 40 years. The mortality rate among the operated patients amounted to 17.6 % [18].

Considering the high risk associated with splenectomy, clinical trials were carried out to decrease spleen size by pharmacological methods. In 1880 Goslin used hypodermic injection of ergotin into the enlarged spleen, and in 1883 Peiper injected fowler’s solution (a solution of potassium arsenite) directly into a leukemic spleen. However, the results of these treatments were not disclosed [8].

Following the example of a wandering kidney, which to the end of the nineteenth century any doctor without a special indication did not want to remove, in 1895 Ludwik Rydygier (1850–1920) operated to attach a wandering spleen to the peritoneum. This surgery, which he called splenopexy (following nephropexy), was based on fixation of the lower end of the spleen in a pocket made in the parietal peritoneum [21]. In the same year Hall carried out splenopexy by making a lumbar incision into the abdomen and fixing the spleen in it by means of tamponade [22]. One of the safest and easiest methods of splenopexy is Bardenheuer’s method, in which the spleen lies with its inferior pole in a retroperitoneal pouch; its pedicle is fixed to the peritoneal wound, and its body is suspended from the tenth rib.

Previous surgery course texts also taught splenorrhaphy, which is the suturing of the spleen for any purpose [23]. At the beginning of the twentieth century, splenopexy became a standard surgical procedure in the treatment of wandering spleen, unless torsion of the pedicle of a wandering spleen was diagnosed [24].

## The impact of experimental physiology on spleen surgery

Attempts to explain the spleen’s importance for life were made by conducting physiological experiments on animals. In 1735 Deisch proved that dogs could live even though they had their spleens removed. He removed the spleen by means of various surgical techniques, which in his opinion would become useful in the clinical setting [8]. Special attention should be paid to pharmacological experiments done in the nineteenth century by Nivet (1838) and Pages (1846), who proved that the spleen decreased its size under the influence of alcohol and quinine. At the same time, similar experiments were done by Kűchenmeister, who used quinine and gentian violet to observe the spleen’s changes in size and the uptake of the dye. These more and more courageous experiments led to braver and braver conclusions. In 1857 John Harley announced that rats could live without a spleen and adrenal glands, and in 1866 Philipeaux presented the successful results of experiments related to transplanting a spleen taken from young animals and then replacing it in the abdominal cavity. The possibility of a spleen being transplanted was confirmed by Tizzoni in 1883 [8].

Such extraordinary spleen characteristics led Erwin Payr (1871–1946) to become interested in the spleen. In 1906 he presented to the German Surgeons’ Congress the results of experiments which referred to implanting fragments of the thyroid gland into a pouch made in the spleen into animals surgically deprived of thyroid; next, omentum was stitched over the splenic wound. Several days later the animals in the control group had their spleen with implemented fragments of thyroid gland removed, which resulted in tetanus and immediate death. Payr used these observations to treat a six-year-old girl diagnosed as a cretin and unsuccessfully treated her with thyroid tablets. He then took a sample of thyroid from the girl’s healthy mother and placed it in the girl’s spleen, which resulted in a considerable improvement in the child’s health. Similar experiments were carried out by William Halsted (1852–1922) [23].

These experiments were of clinical importance, yet determination of the function of the spleen remained unresolved (Fig. 2). This impasse was broken in 1933 when Jűrgen Aschoff (1818–1896) and Edmund Landau (1877–1938) published their work about the reticuloendothelial system. The work indicated anatomical and physiological spleen similarity to Kupffer cells (some macrophages) of the liver, the medullary tissue of the bones, the lymphatic glands, and the cortex of the adrenal gland. All these tissues are composed of similar cells, and after spleen removal they can take over the spleen’s functions, which provides an explanation for the mechanism of how an organism adapts to the results of splenectomy [25].

Aschoff and Landau’s work was of significant value for surgeons who for many years had been observing the fact that a relatively small amount of postoperative bleeding followed removal of the spleen. The examination of blood composition before and after splenectomy indicated a significant postoperative increase in the number erythrocytes and blood platelets. Spleen surgery turned out to be an underlying topic at the International Surgical Congress held in Rome in 1926. Discussions started about accessory spleens, which appeared to number several hundred in one patient, some of them the size of a walnut. Patients who underwent reoperation after splenectomy sometimes had a diagnosis of enlarged splenules; what is more, it was proved that splenules took over the functions of a removed spleen [25].

## Medical technologies and spleen surgery

The introduction of medical technologies represented a watershed in surgery for wandering spleen, which is confirmed by analyses of the PubMed database. Before the era of such medical technologies as isotopic imaging and angiography, the diagnosis of the torsion of a wandering spleen was difficult. Hence, in 1925–1976, publications related to this issue came out on average 0.34 times a year. In 1977–1997 publications referring to wandering spleen torsion appeared on average 3.45 times a year, and by 1998–2011, when laparoscopy became the gold standard in wandering spleen surgery, publications increased to 8.42 times per year.

Twentieth century medicine described wandering spleen in patients ranging from 3 months to 82 years [7]. Accessory spleens occur in 10–15 % of the population. Generally, they are situated close to the splenic hilum, out of which 1–2 % are in the pancreatic tail, which is commonly mistaken on imaging studies for a neuroendocrine tumor [26] or neoplasm [27]. They are also accidentally discovered during surgical procedures conducted on kidneys, the peritoneum, or reproductive organs [28], yet they are most commonly found in the vicinity of the stomach [29]. Thus, surgery contributes to the progress of knowledge about spleen diseases.

At present wandering spleen is defined as an ectopic spleen that moved from its normal anatomical location because of congenital anomalies of the dorsal mesogastrium and the absence or malformation of normal splenic suspensory ligaments [30]. Various imaging techniques can be used to diagnose a wandering spleen. For example, plain radiography, barium enema, scintigraphy, grey-scale sonography, computed tomography (CT), Doppler ultrasonography, and angiography [31, 32]. In cases where CT indicates the absence of the spleen in the left subphrenic space and finds a splenic-like mass in the abdomen or pelvis, diagnosis of a wandering spleen should be kept in mind [33].

The symptoms of abnormal spleen location with torsioned pedicle are splenomegaly and hemoperitoneum. Immediate splenectomy offers very good results in this a life-threatening condition, which occurs in no more than 0.2 % of cases [34]. Since 1998, laparoscopic exploration of the abdominal cavity offers the ultimate diagnostic confirmation [34], enables splenectomy with short postoperative hospital stay followed by a quick recovery. Imaging techniques can be useful in making the right diagnosis, especially angio-spiral CT and color-flow ultrasonography [35].

Since 2000, splenopexy has been carried out laparoscopically if the wandering spleen is healthy and noninfarcted, is of normal size, and has no signs of hypersplenism. The literature describes the sandwich technique, in which two meshes are used to sandwich the spleen [36].

To 2007 the literature indicates almost 500 cases of wandering spleen [36] diagnosed in patients ranging from 3 months to 82 years of age [7]. In recent years special attention has been paid to difficulties related to diagnosing wandering spleen in children [37], because clinical symptoms as well as laboratory test results are atypical. Thrombocytopenia is a rare complication of wandering spleen, usually accompanying torsion of an elongated splenic pedicle [38, 39]. The diagnosis of a wandering spleen can sometimes be very difficult because of the similarity of the clinical symptoms to those of recurrent pancreatitis [40]. Moreover, medical technologies have elucidated the condition known as polysplenia, a complex congenital syndrome associating visceral heterotaxis and concomitant bilateral left-sidedness, when a spleen is divided into several splenules of the same size [41].
